# Case Studies in Physical Therapy: Transitioning A “Hands-On” Approach into A Virtual Platform

**DOI:** 10.5195/ijt.2018.6253

**Published:** 2018-08-03

**Authors:** AIDEEN TURNER

**Affiliations:** 1 VIRTUAL PHYSICAL THERAPISTS, PALM BEACH GARDENS, FLORIDA, USA

**Keywords:** McKenzie MDT, Musculoskeletal, Physical Therapy, Telerehabilitation

## Abstract

Technology is expanding at an unprecedented rate. Because patients value the speed and convenience of the internet, there is an increasing demand for telemedicine. Practitioners must therefore adapt their clinical skills to evolving online technologies. This paper presents a series of three case studies in which a physical therapist first assessed and treated musculoskeletal disorders via a live, secure video. The basis of the mechanical assessment was observation of movement rather than palpation. In each case, the virtual mechanical assessment identified a specific sub-classification with a directional preference. All patients reported improvements in symptoms and function in less than four visits and all maintained a reduction in symptoms after three months. Given the “hands-off” role of the evaluator, this approach can become an effective tool in the evolving healthcare platform of telerehabilitation.

Technology is moving forward at an unprecedented rate, changing how people manage tasks in their daily lives. These changes are naturally migrating into healthcare. Improvements in internet speed and accessibility have resulted in the expansion of digital technologies. These advances have enabled the rise of telemedicine, defined as “the remote delivery of healthcare services and clinical information using telecommunications technology” (American Telemedicine Association, 2018). Telemedicine has paved the way for potentially changing how practitioners deliver quality care, by making it faster, more convenient, and less expensive than traditional office visits and emergency room care (Rajda, 2017). The number of Americans with internet accessibility continues to grow, with $500 million allocated by US Executive Order to extend broadband into rural communities (Reardon, 2018). With improved accessibility, geography will no longer pose barriers to specialty clinicians. Telemedicine is now a service offered by many hospitals, medical specialists, home health agencies, private physicians, and workplaces (American Telemedicine Association, 2018). Patients can now have access to healthcare 24/7, in the convenience of their own homes.

Medicaid has acknowledged the cost-effectiveness of telemedicine: “States are encouraged to use the flexibility inherent in federal law to create innovative payment methodologies for services that incorporate telemedicine technology” (Medicaid, 2017). Most large commercial insurances, including Blue Cross, Aetna, United Healthcare, and Cigna have added telemedicine to their benefits, because it enables improved access to specialty health, and results in a significant reduction in costs (Rajda, 2017; UnitedHealthcare, 2017; Managed Healthcare Executive, 2015; BCBS, 2018; Cigna, 2016). Industry leaders predict that by 2025, over 78 million people worldwide will be using home health technologies and the market will reach $19.5 billion (Landi, 2018).

Advances in technology will require changes in how we assess, obtain data, and manage patients. Patients now expect convenient online access and services. A Harris Poll commissioned by American Well found that 50 million Americans would be willing to switch their primary care physicians (PCPs) to another provider in their area that offers video consultations (American Well, 2017).

Physical therapy is widely regarded as a “hands-on” treatment approach. Currently, the practice of physical therapy requires tedious intake paperwork, manual evaluations, and treatments. To keep up with the technology and demands of the public for faster and more convenient care, the functional assessment tools, evaluations and home instruction must be more efficient without losing integrity (Deloitte Development LLC, 2016). Clinicians must change the very foundation of how they have traditionally operated.

The McKenzie Method ® of mechanical diagnosis and therapy (MDT) is a well-established (trademarked) system for evaluating and treating musculoskeletal (MSK) conditions (McKenzie Institute International, 2018). There is a standardized certification process leading to credentialing (Cert. MDT) and a higher diploma in mechanical diagnosis and therapy (Dip. MDT). Evidence supports the reliability of the MDT assessment for the lumbar spine (Fritz, 2000; Razmjou, 2000; Kilpikpski, 2002; Clare, 2005), cervical (Clare, 2005) as well as the extremities (Takasaki, 2017; Willis, 2017; May, 2009; Abady, 2014).

The MDT methodology utilizes movements performed to end range (i.e., the end of the physiologic range of motion) while evaluating symptomatic and mechanical responses. The response to movement then places the condition into one of four types: derangement, dysfunction, postural, and other (McKenzie, 1981; McKenzie, 1990; McKenzie, 2003) (see Table 1).

The MDT method is generally a “hands-off” approach in favor of empowering the patient. Thus, the approach may be a good fit in the telehealth model of musculoskeletal care. This paper presents case studies that illustrate how physical therapy can transition into a virtual world.

## CASE DESCRIPTIONS

The practitioner was a certified MDT clinician with 20+ years of experience. The mechanical assessment was performed, via a live two-way video. Range of motion was quantified by nil, minimal, moderate, or major loss and a directional preference was established if repetitive movement in one direction had a positive and lasting effect on symptoms, ROM and/or function (McKenzie, 1981; McKenzie, 1990; McKenzie, 2003).

The virtual consultation employed an encrypted, HIPAA compliant application that patients first downloaded onto their smart device. A licensed, internet based real-time communication (iRTC) video streaming was used that is located on a private cloud to maintain security. The security protocol included network and web application firewalls, patient secure login with unique user name/password, and encryption in transit and during sessions with transit layer security (TLS) across all services.

**Figure 1:**
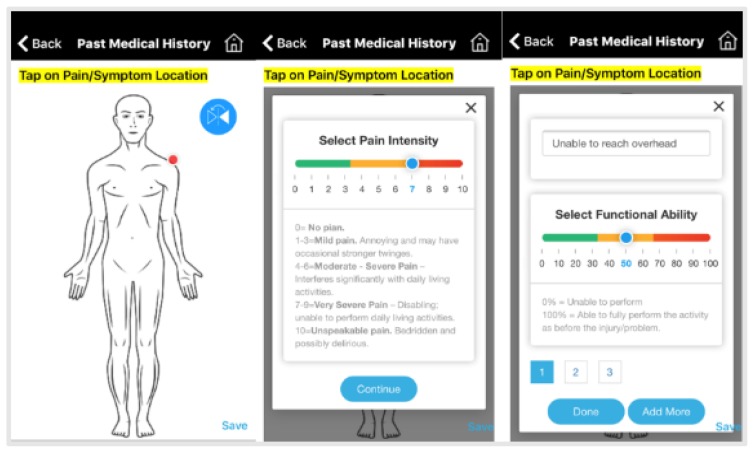
From left to right: Body Pain Diagram (BPD), Numeric Rating Scale (NRS), and Patient Specific Functional Scale.

Consultations occurred via direct access to physical therapy services. Each patient had a smart device and internet connection of at least 1 Megabit per second (Mbps). Before booking an appointment, the patient signed consent, completed Past Medical History (PMH), Numeric Rating Scale (NRS), Body Pain Diagram (BPD) and a Patient Specific Functional Scale (PSFS) (see Figure 1). The patient then scheduled an appointment. The live video session began after both clinician and patient pressed the start button. Patients had access to a full screen video of the clinician on their smart device and a small picture of self, so they could see if their movements were adequately visible during the assessment.

**Figure 2:**
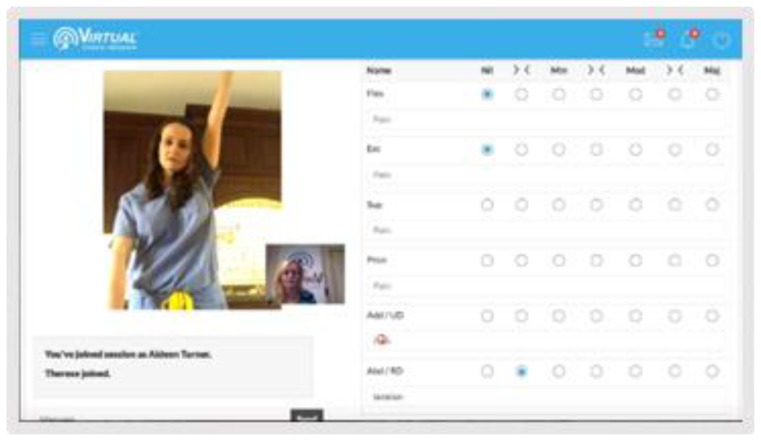
Clinician’s view: The clinician utilizes a computer with left side video and right digital assessment.

The clinician used a laptop computer and a secure internet connection. The left half of the monitor displayed the video conference, and the right side presented a digital assessment (see Figure 2). Before entering into a session, the clinician reviewed the patient’s PMH, and pain/functional questionnaires uploaded to the patient’s profile.

Three patients were selected based on convenience (i.e., the first three to give consent to a virtual case study; availability; and differing body parts). They included individuals with a lumbar, cervical and extremity (elbow) pain.

The initial part of the video consultation required proper placement of the patient’s device so that the clinician would be able to see full movement of the joint being assessed. This usually occurred by resting the smart device on a table/shelf as per the clinician’s instructions for adjustments. The mechanical assessment included questions about the mechanics of symptoms; active range of motion (AROM); repeated movements, and/or sustained postures; and mechanical classification. The consultation also included education and home program instructions with video reference. Follow-up visits were also performed virtually.

Emails were sent 24 hours, 7 days, 4 weeks, and 3 months after the evaluation. The e-mails included a satisfaction rating, and follow-up NRS, BPD, and PSFS. Treatment efficacy was assessed by the number of treatment visits and BPD, NRS and PSFS at pre-assessment and at post-assessment follow-up (i.e., 24 hours and 3 months post evaluation).

### PATIENT #1: LOW BACK PAIN

Patient #1 is a 45-year old male with a long history of low back pain, including an L45 laminectomy in 2008 and L5S1 discectomy in 2011. He had contacted an MDT trained therapist two years prior, secondary to continued low back pain. At that time an assessment revealed an L5 posterior derangement - below the knee, that responded to repeated extension in lying. His symptoms had completely resolved, and he had returned to full function.

He subsequently requested a virtual visit after waking with severe left low back pain and radiation into his left hip and lateral thigh that caused all movement to be very painful. He gave consent for a virtual consultation and case study. He downloaded the app and completed questionnaires. He used a tablet with a cover that converted into a stand. He stated that he was in constant pain, and all movements aggravated his symptoms. He reported that he attempted the exercises given to him two years prior (i.e., lumbar extension in lying). Initially, they provided some relief but the pain had worsened after a few days. At that time, he was unable to work or leave his home, as all movements aggravated his symptoms. He denied foot drop or weakness and his general health was excellent.

#### DAY 1 BASELINES: (TABLES 2 & 3)

**Figure 3:**
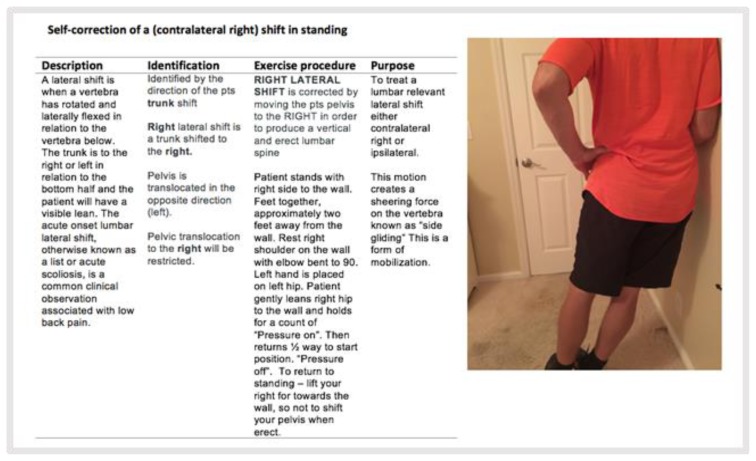
Self-correction of a (contralateral right) shift in standing.

Approximately one minute was spent to enable proper visualization of his lumbar spine, adjusting the placement of the smart device. On observation in standing, a significant right lateral shift was easily seen. AROM revealed a major loss of flexion and extension with right shift and increased pain during motion (PDM). There was a minimal loss of right side glide with increased hip pain, no worse following. Left side glide had a major loss of motion that caused increased low back and thigh symptoms, no worse following. Because of the observed lateral pelvic shift that was relevant (ROM assessment found inability of the patient to move out of the shifted position or major loss of movement in the opposite direction), the mechanical protocol is to attempt to correct the shift (McKenzie 1981). Repeated movements of left side glide were performed against a wall (see Figure 3). The patient adjusted his tablet by rotating it 180 degrees, for proper viewing of the patient against the wall. The patient’s distal symptoms initially increased, so he was instructed to flex his spine slightly forward, while performing the side glide repetitive movement. The mechanical effect after this repeated movement was “Better” or improvement, as symptoms centralized to left low back and side glide ROM increased.

#### INTERVENTION, FOLLOW-UP, AND OUTCOME

The mechanical assessment revealed a directional preference in the sagittal plane with the centralization of symptoms and improvement in ROM. Based on this and the location of symptoms, the patient was classified as having a left L5 derangement (above the knee) with a relevant shift that responded to repeated side glides in standing. He was given left side glides, ten times every two hours for his home exercise program as outlined in the McKenzie original text (McKenzie, 1981) along with a video download for reference. Education on centralization vs. peripheralization; better/worse response to the home exercises; proper standing posture with equal weight bearing on both feet; and proper sitting and sleeping postures were reviewed.

#### DAY 2 (2^ND^ VIRTUAL VISIT) BASELINES (TABLES 2 & 3)

A virtual reassessment the next day revealed that his symptoms centralized to his hip/low back and reduced from PQ 5 to 2/10. The patient already had his smart tablet set-up and was ready for proper viewing when the virtual session began. Upon observation, there was no lumbar shift visible and his AROM improved with only minimal loss of flexion, moderate loss of extension and left side glide. Repeated left side glides movements against a wall improved his overall presentation or “Better” with increased ROM in all directions and pain centralized to low back (PQ 1/10). His mechanical diagnosis was reaffirmed, and the patient was instructed to continue with left side glides in standing (10× every 2 hours) and was again instructed on the importance of proper posture.

#### DAY 5 (3RD VIRTUAL VISIT) BASELINES (TABLES 2 & 3)

Patient reported that he was feeling 90% better. His only difficulty was prolonged sitting, and he continued to avoid any heavy lifting. A reassessment revealed no observed shift and full ROM, except for a minimal loss of left side glide. Repeated movements of left side glide in standing, showed a positive mechanical response of regaining full motion, thus “Better” as a result. The patient was instructed to continue with left side glides for his home program and to avoid a right shift position in sitting and standing.

#### DAY 8 (4^TH^ VIRTUAL VISIT) BASELINES (TABLES 2 & 3)

The patient reported that he was feeling significantly better with 100% functional ability and had no pain other than occasional stiffness. A reassessment performed revealed full ROM except for nil/min loss of left side glide. Repeated flexion had a “Worse” mechanical response causing increased loss of left side glide. Repeated extension had no effect. Repeated side glide in standing produced a “Better” mechanical response of regaining full left side glide motion. The patient was again instructed to continue with left side glides in standing for his HEP (10× every 2 hours). He was also instructed on the recovery of function to begin after regaining the ability to repetitively flex without loss of side glide motion.

The patient followed up by e-mail on Day 15. He wrote that he was feeling 100% symptom-free. Instruction was provided by e-mail to begin a trial of flexion: first 10 times and then to check his ROM, particularly side glide. Then 30 times and check ROM. If there was no loss of ROM, he was stable to return to full function and to add 10 flexion/day for HEP and to check ROM regularly. If there was any loss of motion, he was instructed to contact the provider by e-mail or a virtual visit.

A standard 4-week e-mail questionnaire was sent asking about satisfaction, pain, and function, but there was no response. Another follow-up e-mail was sent at three months. The patient wrote that he continued to be symptom-free and had no functional limitations. He noted that he was pleased with his virtual rehabilitation and would opt for that platform in the future, because of the convenience and avoiding a 45-minute drive each way.

### PATIENT #2: RIGHT CERVICAL PAIN

Patient #2 was a 49-year old female with a busy work schedule. She contacted our office and had trouble scheduling her evaluation, because of her long work hours. She was asked if she would like to try a virtual consultation and she quickly agreed. She was given instructions on how to download the app and what to expect. Her virtual visit was scheduled for later that afternoon. She used her smartphone and had no prior experience with a mechanical assessment or physical therapy. She was in excellent health and reported an insidious onset of right cervical/upper trapezius pain for two months. She noted occupational stress of computer work and was a leisure golfer. She denied any upper extremity symptoms (see Table 4).

She initially had some difficulty setting up her smartphone. She had it leaning on a stack of folders, but it slipped forward until she found a small box of paperclips to put in front of her phone. She was observed sitting in poor posture with a forward head. There were no observable deviations or abnormalities. Posture correction decreased symptoms from 3/10-1/10. Assessment of her AROM revealed a moderate loss of retraction, extension and right rotation. Repeated movements of retraction/extension produced a “Better” mechanical response of increased ROM and decreased symptoms (PQ 0.5/10) (see Table 5).

#### INTERVENTION, FOLLOW-UP, AND OUTCOME

**Figure 4:**
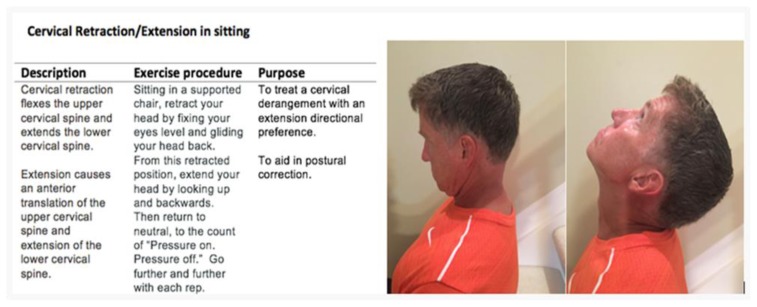
Cervical retraction/extension in sitting.

The mechanical response to posture correction, reduction of symptoms and increased ROM following repeated movements, revealed a posterior cervical derangement (above the elbow) that responded to repeated retraction/extension. Figure 4 demonstrates cervical retraction/extension in sitting.

The patient was given education on proper sitting with a lumbar roll and a home program regimen of retraction/extension 10× every 2 hours with video reference.

#### DAY 3 (2^ND^ VIRTUAL VISIT) BASELINES: TABLES 4 & 5

The patient reported that she was feeling 75% better and had bought a phone holder for the video session. She continued to have poor sitting posture. A reassessment revealed nil/minimal loss of extension and right rotation. Repeated movements of retraction/extension produced a “Better” mechanical response of full extension and right rotation ROM, which affirmed the preliminary diagnosis. The patient was instructed to continue with the current exercise program. The practitioner reviewed the importance of proper sitting and provided an explanation for the recovery of function.

#### DAY 7 BASELINES (FOLLOW-UP BY EMAIL): TABLES 4 & 5

The patient replied by e-mail that she had no pain and was feeling 100%. She wrote that she continued to perform her exercises and was much more aware of her posture in sitting. The instruction was given on recovery of function and to contact the practitioner if there was any loss in her ROM.

#### 4 WEEKS AND 3 MONTH BASELINES (EMAIL FOLLOW-UP): TABLES 4 & 5

The patient replied that she continued to feel 100% symptom free. She expressed that she was very satisfied with her virtual rehabilitation and would choose it again because it was convenient to her busy schedule and provided knowledge and explanation of her ailment.

### PATIENT #3: RIGHT LATERAL ELBOW PAIN

Patient #3 was a 50-year old active male with complaints of right lateral elbow pain that he attributed to weight lifting. He had contacted our office and agreed to a virtual consultation and case study. He downloaded the app onto his smartphone and completed the PMH, Pain and Functional questionnaires and initiated the virtual visit. He reported that his symptoms started approximately six weeks prior and were brought on only by the performance of elbow curls and single dumbbell row. Otherwise, he noted only stiffness and a need to “move his elbow.” The clinician gave him instruction on placing his smartphone (the stand was already attached) on the table in front of him so that she was able to see the movement of his elbow. Adjustments were then made by the patient when he saw his elbow go off the frame when moving it during the assessment.

#### DAY 1 BASELINES: TABLES 6 &7

Upon observation, there did not appear to be any abnormalities. He had full flexion AROM, but pain at end range and a minimal loss of extension with pain at end range. Self-administered isometric of wrist/middle finger extension was painful and weak. Repeated movements of elbow extension with overpressure in weight bearing produced a “Better” response with increased extension ROM, decreased pain with end range flexion/extension and decreased pain with wrist/finger extension isometri (see Figure 4).

#### INTERVENTION, FOLLOW-UP AND OUTCOME

Based on the mechanical response to repeated elbow extension improving his symptoms and ROM, this patient was classified as having an elbow derangement. The patient was instructed in better/worse response to exercise and to minimize flexion movements temporarily. He was given elbow extension with overpressure to perform 10 times every 3 hours for his home program with video reference.

#### 24 HOUR BASELINE (E-MAIL FOLLOWUP): TABLES 6 & 7

The patient wrote that he had not yet returned to weight lifting, so his PSFS was left the same since he was unable to assess at that time. He reported that he did feel immediate relief following performance of home exercise. The patient was instructed to continue with the same home program and to follow-up in a few days.

#### DAY 7 BASELINE (E-MAIL FOLLOW-UP): TABLES 6 & 7

The patient wrote that he was feeling 75% better overall. He noted that he continued to intermittently have discomfort when lifting weights and sometimes at work, but the performance of elbow extension with overpressure gave immediate relief. He was instructed to continue with the HEP and to follow-up with any changes.

#### ONE MONTH BASELINE (E-MAIL FOLLOW-UP): TABLES 6 &7

The patient reported feeling 95% relief, noting only occasional discomfort that was relieved immediately with elbow extension with overpressure. He was given explanation on recovery of function and to continue with his current HEP.

#### 3 MONTH BASELINE (E-MAIL FOLLOWUP): TABLES 6 &7

The patient wrote that he has been symptom free and had returned to his full work-outs with no pain. He rated very high satisfaction for his virtual consultation, because of ease, clear explanations and knowledge of the clinician.

## DISCUSSION

The purpose of this paper was to introduce the ability to assess MSDs virtually, via MDT’s mechanical assessment. In all three cases, pain was eliminated and the patients returned to full function and maintained full status three months later. The NPS, BPD, and PSFS were used to monitor symptom response and functional gains. All three patients rated their satisfaction with the experience as high, noting convenience, communication, knowledge of the clinician, and clear explanations, as reasons for the excellent experience. The number of visits, abolishment of symptoms, return to full function and maintenance of this status, introduce the potential of utilizing MDT to enable telerehabilitation.

The patients were not randomly selected and the sample size was small due to the nascent status of telerehabilitation. Though this case series lacks experimental controls, it introduces the possibility of being able to provide a sufficient musculoskeletal assessment virtually.

Not every patient will be a candidate for virtual assessment/treatment. The following limitations could contraindicate a virtual approach:

1. **Patient technology challenges:** The patient must be familiar with downloading an app and navigating through the application’s features.
2. **Connectivity challenges**: Poor or no internet connectivity would contraindicate a virtual approach.
3. **“Hands-on” approach needed**: Some patients will require a “hands-on” approach, such as a manual therapy for a shift correction, overpressure for treatment, manual contact for guidance or balance, etc.
4. **Neurological assessment needs:** The practitioner will be unable to perform a full neurological assessment, specifically DTRs.
5. **Strength testing limitations:** The practitioner must modify strength testing, relying solely on the patient. Strength can be performed by isometrics, self-manual resistance or functional activities such as knee dips for quad strength.

Future research requires larger sample sizes and randomized groups. Different assessment strategies need to be explored on performing neuro screens, strength and balance assessments virtually. Though not every patient is suited for telerehabilitation, in time, many more will embrace this new service delivery model.

## CONCLUSION

Technology is moving forward at an unprecedented rate. It is changing how we do things in our daily lives, and these changes are naturally migrating into healthcare. To stay current, clinicians will need to adapt their skills to meet the demands of the public for more convenient, faster, better, and cheaper access to specialty care.

There is now the potential to employ technology to reach more individuals with musculoskeletal disorders (MSDs) and consequently to empower patients to take charge of their health, reduce costs, improve convenience, increase accessibility to MSD specialists in rural areas, and improve outcomes. The purpose of this paper was to demonstrate that it is possible to perform a virtual musculoskeletal assessment. Further research is required with larger sample sizes, as well as to develop novel ways to virtually assess strength, neurological signs, balance and conduct special tests.

## Figures and Tables

**Table 1:**
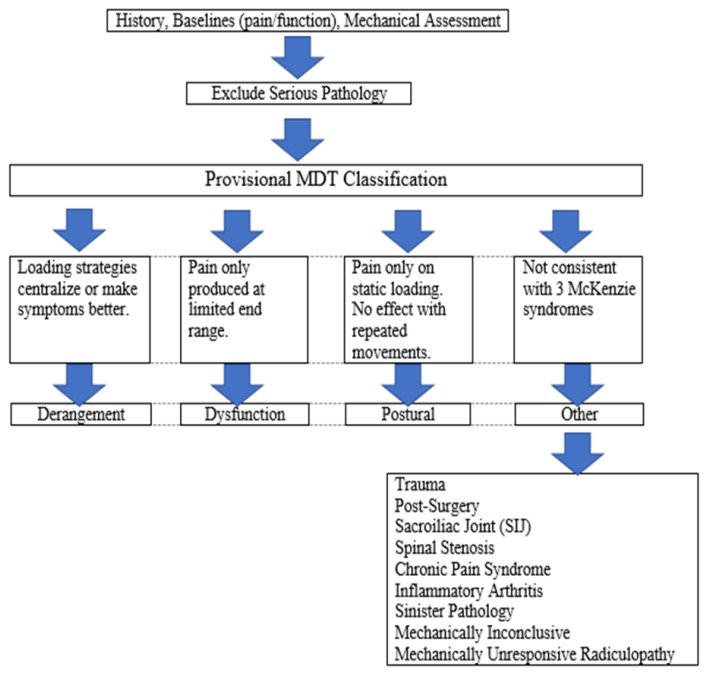
McKenzie MDT Classification System

**Table 2:**
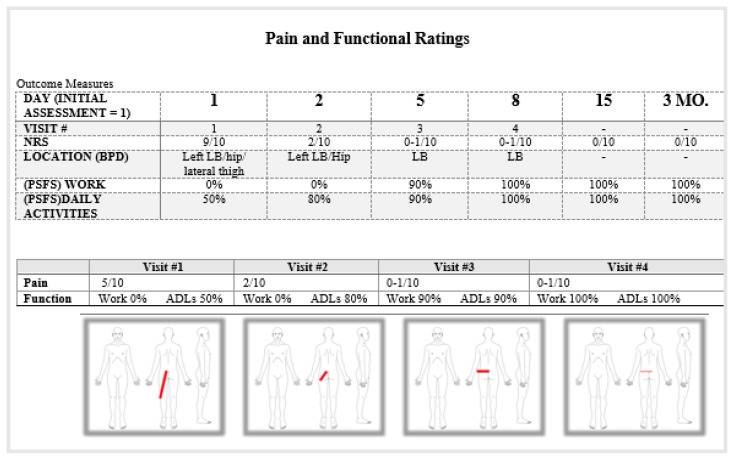
Patient #1 - Low Back and Left Hip/Lateral Thigh Pain

**Table 3:** Patient #1- Low Back and Left Hip/Lateral Thigh Pain

Mechanical Assessment			

Active Range of Motion and Repeated Movements of the Low Back			
Day 1 / Visit 1 Motion	AROM	Response after 1 rep	Repeated movement response

Flexion	Major loss/(+) R shift	Increased PDM	NT

Extension	Major loss/(+) R shift	Increased PDM	NT

R Side Glide	Minimal loss	Increased distal sx	NT

L Side Glide	Major loss	Increased PDM	Better – Centralized to L LB and Inc ROM/Decreased shift

**Day 2/Visit 2**			

Flexion	Minimal loss	Increased PDM	NT

Extension	Moderate loss	Increased PDM	NT

R Side Glide	No loss	NE	NT

L Side Glide	Moderate loss	Decreased	Better with – Centralized to LB and increased ROM

**Day 5 / Visit 3**			

Flexion	No loss	NE	NT

Extension	Minimal loss	NE	NT

R Side glide	No loss	NE	NT

L Side glide	Minimal loss	ERP	Better with full ROM

**Day 8 / Visit 4**			

Flexion	No loss	NE	Worse – Loss of L SG motion

Extension	No loss	NE	Worse – PDM

*Note.* PDM = pain during motion; ERP = end range pain; NE = no effect; NT = not tested

**Table 4:**
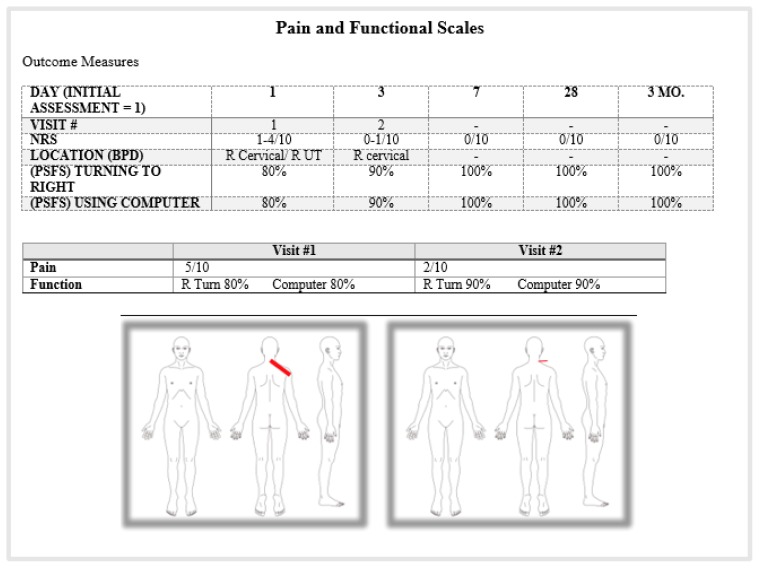
Patient #2 - Right Cervical and Right Upper Trap Pain

**Table 5:** Patient #2 - Right Cervical and Upper Trapezius Pain

Mechanical Assessment: Active Range of Motion and Repeated Movements of the Cervical Spine			

Day 1/Visit 1 Motion	AROM	Response after 1 rep	Repeated movement response
Protrusion	Full	Increased ERP	Worse - Increased pain
Flexion	Full	Increased PDM	Worse – Increased pain
Retraction	Moderate loss	Increased distal sx	Better – Increased ROM/Decreased pain
Extension	Moderate Loss	Increased PDM	Better – Increased ROM/Decreased pain (0.5/10)
R Lateral Flex	Min/mod loss	Increased PDM	NT
L Lateral Flex	Minimal loss	Increased PDM	NT
R Rotation	Moderate loss	Increased PDM	NT
L Rotation	Minimal loss	Increased PDM	NT

**Day 3/Visit 2**			

Protrusion	Full	NE	NE
Flexion	Full	NE	Worse – Produced right cervical pain
Retraction	Full	NE	Better – Decreased pain
Extension	Nil/minimal Loss	Increased PDM	Better – Increased ROM/Abolished pain
R Lateral Flex	Full	NT	NT
L Lateral Flex	Full	NT	NT
R Rotation	Nil/minimal loss	NT	NT
L Rotation	Full	NT	NT
R Side glide	Full	NT	NT
L Side glide	Full	NT	NT

**Table 6:**
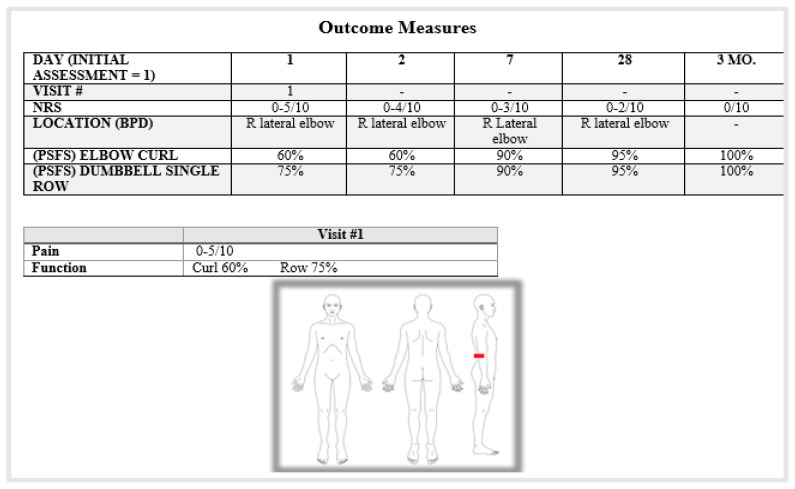
Patient # 3: Right Lateral Elbow Pain

**Table #7:** Patient #3 - Right Lateral Elbow Pain

Mechanical Assessment: Active Range of Motion and Repeated Movements of the Right Elbow			
Day 1/Visit 1 Motion	AROM	Response after 1 rep	Repeated movement response
Flexion	Full	ERP	Worse – Produced pain
Extension	Minimal loss	ERP	Worse – Increased pain
Pronation	No loss	NE	NT
Supination	No loss	ERP	NT
Isometric R wrist/middle finger extension		Pain and weakness	

Note. PDM = pain during motion; ERP = end range pain; No Effect = NE; Not Tested = NT
